# MiR‐122 modification enhances the therapeutic efficacy of adipose tissue‐derived mesenchymal stem cells against liver fibrosis

**DOI:** 10.1111/jcmm.13208

**Published:** 2017-05-24

**Authors:** Guohua Lou, Ying Yang, Feifei Liu, Bingjue Ye, Zhi Chen, Min Zheng, Yanning Liu

**Affiliations:** ^1^ Collaborative Innovation Center for Diagnosis and Treatment of Infectious Diseases State Key Laboratory for Diagnosis and Treatment of Infectious Diseases The First Affiliated Hospital School of Medicine Zhejiang University Hangzhou China

**Keywords:** adipose tissue‐derived mesenchymal stem cells, miR‐122, hepatic stellate cells, exosome, liver fibrosis

## Abstract

Mesenchymal stem cell (MSC) transplantation alone may be insufficient for treatment of liver fibrosis because of complicated histopathological changes in the liver. Given that miR‐122 plays an essential role in liver fibrosis by negatively regulating the proliferation and transactivation of hepatic stellate cells (HSCs), this study investigated whether miR‐122 modification can improve the therapeutic efficacy of adipose tissue‐derived MSCs in treating liver fibrosis. MiR‐122‐modified AMSCs (AMSC‐122) were constructed through lentivirus‐mediated transfer of pre‐miR‐122. MiR‐122‐modified AMSCs expressed high level of miR‐122, while they retained their phenotype and differentiation potential as naïve AMSCs. AMSC‐122 more effectively suppressed the proliferation of and collagen maturation in HSCs than scramble miRNA‐modified AMSCs. In addition, AMSC‐derived exosomes mediated the miR‐122 communication between AMSCs and HSCs, further affecting the expression levels of miR‐122 target genes, such as insulin‐like growth factor receptor 1 (IGF1R), Cyclin G(1) (CCNG1) and prolyl‐4‐hydroxylase α1 (P4HA1), which are involved in proliferation of and collagen maturation in HSCs. Moreover, miR‐122 modification enhanced the therapeutic efficacy of AMSCs in the treatment of carbon tetrachloride (CCl_4_)‐induced liver fibrosis by suppressing the activation of HSCs and alleviating collagen deposition. Results demonstrate that miR‐122 modification improves the therapeutic efficacy of AMSCs through exosome‐mediated miR‐122 communication; thus, miR‐122 modification is a new potential strategy for treatment of liver fibrosis.

## Introduction

Transplantation of bone marrow mesenchymal stem cells (MSCs) is an alternative treatment of liver cirrhosis 1, an irreversible disease that may eventually lead to liver cancer. As adipose tissue is an accessible source of cells, adipose tissue‐derived MSCs (AMSCs) are currently being investigated 2. A sufficient number of cells for stem cell‐based therapy can be obtained non‐invasively from this tissue without damaging a patient's health. These cells present very promising applications in therapy of liver diseases. Studies have suggested that AMSCs display a special affinity to hepatocyte differentiation *in vitro* and liver regeneration *in vivo* 2, 3. Thus, AMSCs are very attractive tool for future stem cell therapy of liver diseases.

However, some studies have shown that many MSCs recruited into a fibrotic liver have become myofibroblasts, and at least some MSCs likely contribute to wound‐healing mechanisms and fibrogenesis after liver parenchymal cell death 4. Thus, genetic manipulation that induces MSCs to locally produce therapeutic genes possibly resulted in their altered cell fates and in their enhanced therapeutic abilities.

Liver cirrhosis is the result of pathological response to initial liver injuries of various causes. Hepatic stellate cells (HSCs), which play a key role in liver fibrogenesis, are well documented 5. However, the mechanisms of pathogenesis and progression of liver fibrosis are complicated and may vary depending on the aetiology of the disease. Increasing amount of evidence suggests that certain miRNAs are critically involved in different developmental stages of liver fibrosis, including activation of HSCs and production of ECM proteins 6, 7. Bing the most abundant miRNA in a normal liver, miR‐122 is reduced in advanced liver diseases, such as cirrhosis and hepatocellular carcinoma 8, 9. Mice with hepatocytes wherein miR‐122 was selectively deleted displayed altered lipid metabolism, hepatic inflammation, fibrosis and high incidence of hepatocellular carcinoma 10. Recent studies have suggested that miR‐122 negatively regulates collagen production in HSCs 11, 12. Thus, targeted delivery of miR‐122 to the liver, particularly to the HSCs, is a potential novel therapeutic approach for treatment of liver fibrosis.

In this study, we suggest that the combination of AMSC transplantation and miR‐122 transfer is superior over AMSC transplantation for the treatment of liver fibrosis.

## Materials and methods

### Mice

C57BL/6 mice (6‐week old) were purchased from Zhejiang Academy of Medical Science, Hangzhou, Zhejiang, China. The animals were reared under a specific pathogen‐free condition, and all procedures were reviewed and approved by the Institutional Animal Care and Use Committee of Zhejiang University.

### HSC isolation

Primary mouse HSCs were isolated as previously described 13 and cultured in Dulbecco's modified Eagle's medium (DMEM) containing 10% FBS (Gibco, Carlsbad, CA, USA) and 1% antibiotic–antimycotic (Gibco) under a humidified atmosphere with 5% CO_2_. The primary HSCs used 3 days after isolation were as quiescent cells. HSCs were cultured for 7 days to allow transactivation.

### Cell culture

LX‐2, an immortalized human HSC line, was maintained in DMEM containing 10% FBS and 1% antibiotic–antimycotic. AMSCs isolated from adipose tissues of adult patients undergoing liposuction were denoted as hAMSCs; those isolated from subcutaneous and visceral adipose tissues of C57BL/6 mice were denoted as mAMSCs. AMSCs were maintained in MesenPRO RS™ Medium (Gibco) containing 2 mM l‐glutamine (Gibco) and 1% antibiotic–antimycotic. Experiments were conducted on AMSCs at passages 3‐6.

### Construction of miR‐122‐modified AMSCs

MiR‐122 was overexpressed in AMSCs by using lentivirus (LV)‐mediated transfer of pre‐miR‐122 precursor molecules (LV‐miR‐122). Before infection, 1 × 10^6^ AMSCs were seeded in 10 ml of DMEM supplemented with 10% FBS overnight. AMSCs were subsequently transfected with 100 nM LV‐miR‐122 or LV‐cel‐miR‐67 (Vigene Biosciences, Rockville, MD, USA) at a multiplicity of infection (MOI) of 10 in the presence of polybrene (8 μg/ml; Sigma‐Aldrich, St. Louis, MO, USA) for 24 hrs. Stable transfectants were used in the subsequent experiments.

### Isolation and detection of miRNA

Total RNA enriched with miRNAs was isolated from AMSCs by using a miRVana miRNA isolation kit (Applied Biosystems, Foster City, CA, USA) according to the manufacturer's instructions. Complementary DNA was synthesized from the isolated miRNAs by using TaqMan™ hsa‐miR‐122‐ or mmu‐miR‐122‐specific primers (Applied Biosystems) and TaqMan™ MicroRNA Reverse Transcription Kit (Applied Biosystems). To examine the expression of miR‐122, we performed qPCR by using ABI PRISM7900 (Life Technologies, Waltham, MA, USA) and TaqMan™ Universal Master Mix (Applied Biosystems) according to the manufacturer's instructions. The relative quantity of miR‐122 expression (fold change) was determined using the 2^−ΔΔCT^ method (ΔCt = Ct_miR‐122_ – Ct_U6_ and ΔΔCt = ΔCt_miRNA‐modified AMSC_–ΔCt_naïve AMSC_). U6 was used as endogenous control.

### Flow cytometric analysis of cell immunophenotype

MiRNA‐modified AMSCs were incubated with phycoerythrin (PE)‐labelled primary antibodies raised against CD31, CD45, HLA‐DR, CD29, CD44, CD73, CD90 and CD105 (Becton‐Dickinson, San Jose, CA, USA) at 4°C for 30 min. PE‐labelled IgG1 was used as isotype control. After washing and resuspension in PBS, the samples were analysed by a BD Accuri™ C6 flow cytometer (Becton‐Dickinson).

### Cell cycle analysis

Cells were stained by using a cell cycle staining kit (MultiSciences, Hangzhou, China) according to the manufacturer's instructions. Distribution of cell cycle phases among cells with different DNA contents was determined by using a BD Accuri^®^ C6 flow cytometer.

### Differentiation of AMSCs

Differentiation of miRNA‐modified AMSCs into adipocytes and chondrocytes was determined using a StemPro^®^ adipogenesis and chondrogenesis differentiation kit (Gibco). Oil red O or Alcian Blue staining was subsequently performed to detect adipocytes and chondrocytes, respectively.

### Cell viability

LX‐2 cells (2 × 10^3^) were seeded on 96‐well plates overnight, and then, the culture medium was replaced with the supernatant of AMSCs. Cell proliferation was determined 72 hrs after culture using an MTT assay (Sigma‐Aldrich).

### Cell co‐culture

AMSCs (2 × 10^5^ cells/well) were inoculated in a semi‐permeable membrane (Transwell insert) in the upper part of 6‐well culture plates, and LX2 cells or primary mouse HSCs (2 × 10^5^ cells/well) were inoculated in the lower part to establish a double‐cell co‐culture system. LX2 cells or primary HSCs cultured alone were used as control (untreated, U.T). After co‐culture for 48 hrs, the LX2 cells or primary HSCs were harvested for qPCR and Western blot assay.

### RNA isolation and qPCR

Total RNA was isolated from LX2 cells or mice liver samples by using TRIzol. First‐strand cDNA was subsequently synthesized followed by qPCR analysis by using ABI Prism 7900 (Applied Biosystems) to examine the expression levels of collagen type I alpha 1 (Col1A1), prolyl‐4‐hydroxylase α1 (P4HA1), Cyclin G(1) (CCNG1), insulin‐like growth factor receptor 1 (IGF1R), TGF‐β1 and α‐SMA; GAPDH and β‐actin were used as internal controls. The 2^−ΔΔCT^ method was used to determine the relative mRNA expression levels of these genes.

### Western blot analysis

To determine the protein expression levels, we prepared whole‐cell or tissue extracts, which were fractionated using SDS‐PAGE. After electrophoresis, the proteins were electro‐transferred onto nitrocellulose membranes, blotted with COL1A1 (1:200, Santa Cruz Biotechnology, Dallas, TX, USA), P4HA1 (1:2000; Abcam, Cambridge, CA, USA), CCNG1 (1:1000; Abcam), IGF1R (1:1000; Abcam), TGF‐β1 (1:1000; Cell Signaling Technology, Danvers, MA, USA), α‐SMA (1:2000; Abcam), β‐actin (1:10000, Huabio) and GAPDH (1:10000; Abcam) antibodies. The proteins were finally detected using an enhanced chemiluminescence regent (Amersham, Stavanger, Norway). Blots against β‐actin or GAPDH served as loading controls.

### Exosome preparation and harvest

AMSC‐derived exosomes were isolated and identified as previously described 14. Exosomes were isolated from the supernatant of AMSCs by using an ExoQuick‐TC Kit (System Biosciences, Johnstown, PA, USA) according to the manufacturer's instructions. These exosomes were subsequently characterized through Western blot analysis of exosome surface markers, such as CD9 (1:2000; Abcam) and CD63 (1:2000; Abcam). Precipitates from AMSC supernatant were used as negative control. The protein content of exosomes was determined using a BCA protein assay kit (Pierce, Rockford, IL, USA). Exosome pellets were subsequently resuspended in sterile PBS at a total protein concentration of 5 μg/μl.

### Confocal microscopic studies

AMSCs were labelled with the phospholipid membrane dye lipophilic carbocyanine DilC_16_ (3) (1.25 μM) 15. After 10 min. of incubation at 37°C, the cells were washed and then resuspended in fresh media for 48 hrs. Fluorescent exosomes were collected and added into recipient LX2 cells. Afterwards, the cells were fixed with methanol, mounted on slides, and imaged *via* confocal microscopy (Olympus, Center Valley, PA, USA). Background fluorescence was subtracted using unstained cells.

### AMSC transplantation

Carbon tetrachloride (CCl_4_) (diluted 1:1 in olive oil; Sigma‐Aldrich) was administered through intra‐peritoneal (IP) injection at 1 ml/kg twice per week to induce liver cirrhosis. One day after the fourth injection of CCl_4_, 1 × 10^5^ mAMSC‐122 or mAMSC‐67 or similar volume of saline as control was injected into the tail vein. The mice were further treated with CCl_4_. After 4 weeks, the mice were killed, and blood serum and liver samples were collected to assess the extent of liver fibrosis. Blood serum was evaluated for biochemical parameters. The liver samples were evaluated through histochemical analysis, qPCR and Western blot assay.

### Histological examination

Four weeks after AMSC transplantation, liver samples were harvested 72 hrs after the last injection of CCl_4_, fixed with 10% formalin and embedded with paraffin. Histological analyses of liver tissues were conducted by Sirius red staining to determine the extent of the development of liver fibrosis.

### Evaluation of serum parameters for liver fibrosis and function

Alanine aminotransferase aspartate (ALT) and aspartate aminotransferase (AST) were measured using FUJI DRI‐CHEM Slide GFP/ALT‐PIII and GOT/AST‐PIII, respectively, according to the manufacturer's instructions for DRI‐CHEM 4000ie (FUJIFILM). Serum hyaluronic acid (HA) and procollagen III‐N‐peptide (P‐III‐P) were measured by using radio‐immunoassay.

### Evaluation of hepatic hydroxyproline

Hepatic hydroxyproline content was measured using a hydroxyproline kit (Nanjing Jian Cheng Bioengineering Institute, Nanjing, Jiangsu, China) according to the manufacturer's instructions 16. The hydroxyproline content of the liver was expressed in micrograms per gram of wet weight (μg/g).

### Statistical analysis

Differences between groups were analysed using the conventional Student's *t*‐test or anova. Each experiment was performed at least three times, and data are presented as mean ± S.D. *P* < 0.05 indicated statistical significance.

## Results

### Construction of miR‐122‐modified AMSCs

**Figure 1:**
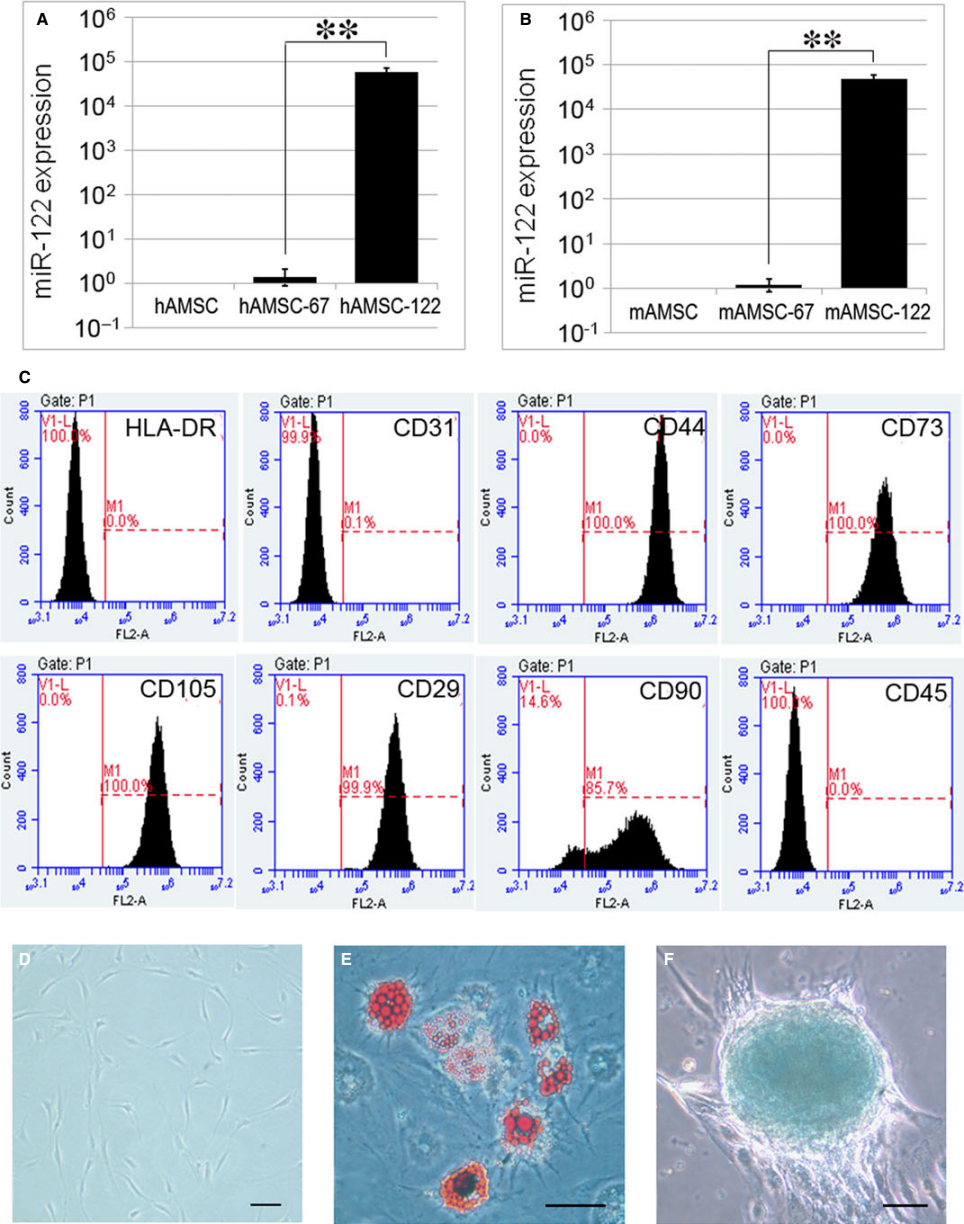
Construction of miR‐122‐modified AMSCs. (**A** and **B**) qPCR detection of miR‐122 expression in hAMSCs (**A**) and mAMSCs (**B**). Expression values were normalized to naïve AMSCs. AMSC‐122: miR‐122‐modified AMSC; AMSC‐67: cel‐miR‐67‐modified AMSC. Data are presented as means ± S.D. (**P < 0.01, *n* = 3) (**C**) Flow cytometric analysis of the surface markers in miR‐122‐modified hAMSCs. (**D**) Cellular morphology of miR‐122‐modified hAMSCs in culture (×200). (**E**) Oil Red O staining of hAMSC‐122 cultured in adipogenesis differentiation medium for 14 days (×400). (**F**) Alcian Blue staining of hAMSC‐122 cultured in chondrogenesis differentiation medium for 21 days (×400). Scale bar = 50 μm.

Mouse or human AMSCs were transfected with LV‐miR‐122 or LV‐cel‐miR‐67, which has no known mRNA binding targets in mouse and human, to serve as control. After the stable transfection, miR‐122 expression in miR‐122‐modified AMSC (AMSC‐122) was assayed by qPCR and compared that in cel‐miR‐67‐transfected AMSCs. MiR‐122 expression was significantly higher in AMSC‐122 than in scramble miRNA‐modified AMSC (AMSC‐67) (Fig. 1A and B).

No differences in phenotype were observed between miRNA‐modified mAMSCs and naïve mAMSCs. They were both negative for CD31, CD45 and I‐A/I‐E‐ and positive for CD29, CD44, CD90.2 and CD105. Moreover, the phenotype of miR‐122‐modified hAMSC was similar to naïve hAMSCs; they were both negative for CD31, CD45 and HLA‐DR and positive for CD29, CD44, CD73, CD90 and CD105 (Fig. 1C).

To further test the differentiation property of miR‐122‐modified AMSCs *in vitro*, multi‐lineage differentiation into adipocytes and chondrocytes were induced, and differentiated cells were detected through Oil red O staining and Alcian blue staining, respectively. As shown in Figure 1E and F, miR‐122‐modified AMSCs can still differentiate into multi‐lineage cells similar to naïve AMSCs.

### MiR‐122‐modified AMSCs effectively suppress the proliferation of and collagen maturation in HSCs

**Figure 2:**
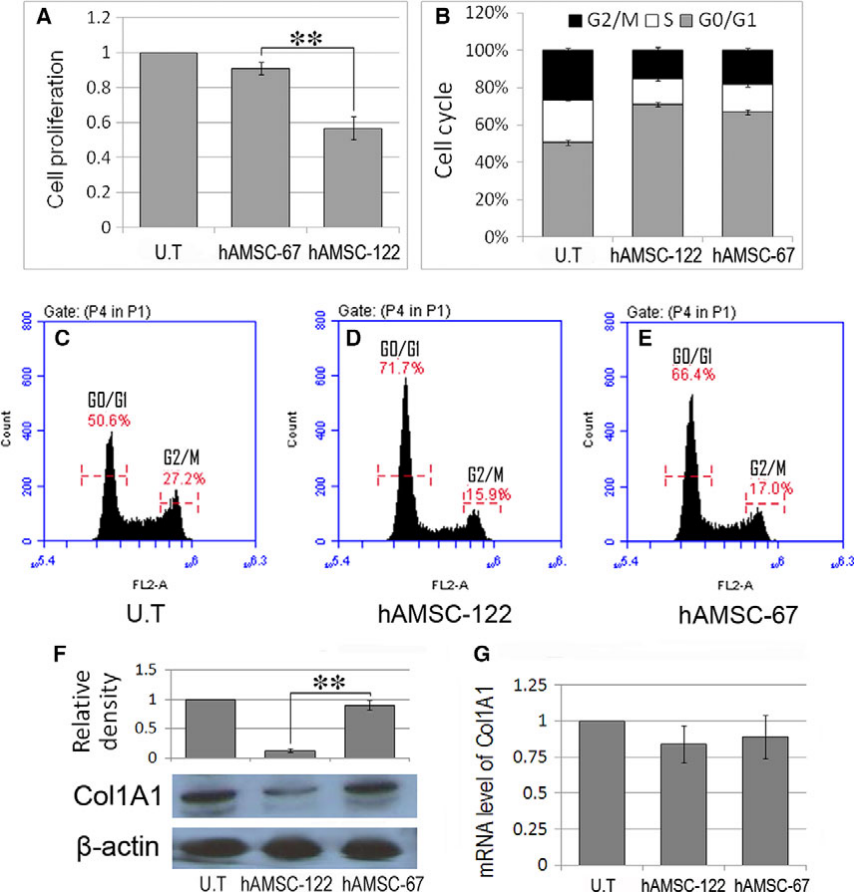
MiR‐122‐modified hAMSCs suppress proliferation of and collagen maturation in hepatic stellate cells (HSC) cell line. (**A**) LX2 cells were cultured with supernatant of hAMSC‐122 or hAMSC‐67. MTT assay was used to examine cell proliferation 3 days after culture. (**B**–**E**) LX2 cells were similarly cultured with the supernatants above. The LX2 cells were stained with PI 24 hrs after culture for cell cycle analysis. (**F** and **G**) LX2 cells were co‐cultured with hAMSC‐122 or hAMSC‐67 for 48 hrs. The protein and mRNA levels of Col1A1 in LX2 cells were analysed by Western blot (**F**) and qPCR (**G**), respectively. Blots against β‐actin served as loading controls. Data are presented as means ± S.D. (**P < 0.01, *n* = 3). U.T, untreated.

Considering that HSCs play a key role in liver fibrogenesis, we evaluated the effect of miR‐122‐modified AMSC on HSCs. As shown in Figure 2A, cell proliferation of LX2 cells treated with the supernatant of hAMSC‐67 was slightly inhibited compared with that of untreated (U.T.) LX2 cells. However, cell growth was significantly inhibited after treatment with the supernatant of hAMSC‐122. Moreover, treatment of LX2 cells with hAMSC‐122 supernatant for 24 hrs increased the percentage of G0/G1 population from 50.6% ± 1.3% to 71.2% ± 1.1%. Treatment of LX2 cells with hAMSC‐67 supernatant also increased the G0/G1 population from 50.6% ± 1.3% to 66.8% ± 1.5% (Fig. 2B–E). We then investigated the effect of miR‐122‐modified AMSCs on Col1A1 expression in LX2 cells. As shown in Figure 2F, the protein expression of mature Col1A1 significantly decreased in hAMSC‐122 co‐cultured LX2 cells compared with that in the hAMSC‐67 co‐cultured group or U.T. LX2 cells. By contrast, co‐culture with hAMSC‐122 exerted no effect on the mRNA level of Col1A1 (Fig. 2G). These results indicated that miR‐122 modification in AMSC inhibited collagen synthesis and maturation.

**Figure 3:**
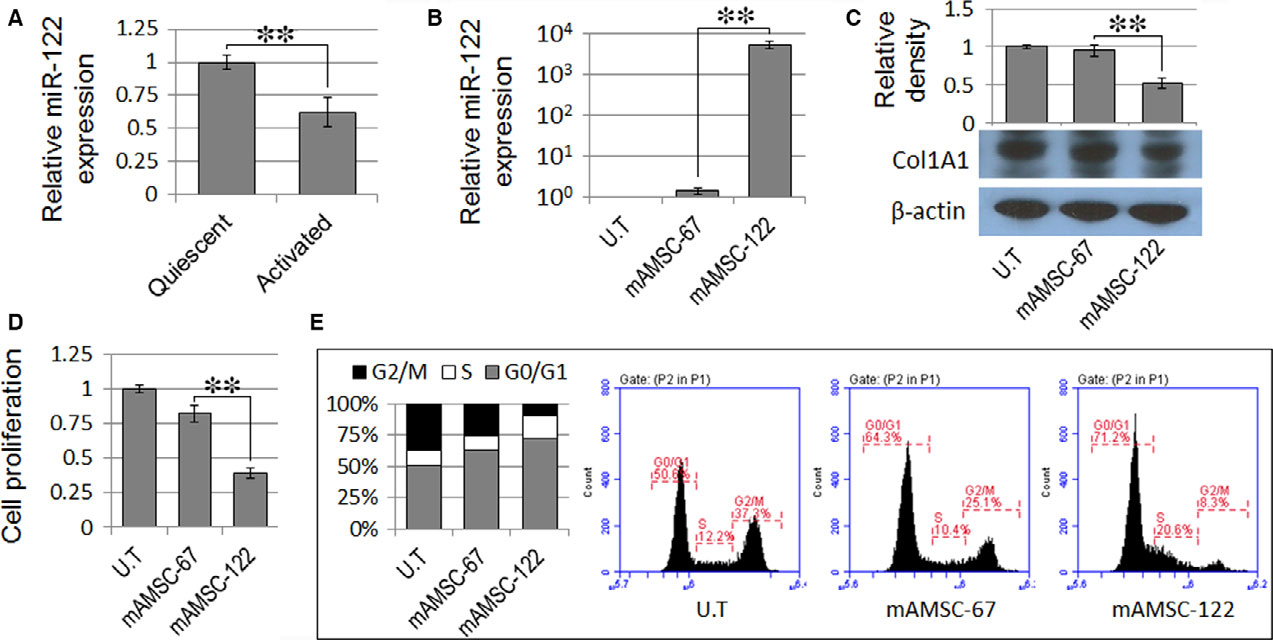
MiR‐122‐modified mAMSCs suppress proliferation of and collagen maturation in primary hepatic stellate cells (HSCs). (**A**) MiR‐122 expression decreased in culture‐activated mouse HSCs. (**B**) MiR‐122 expression increased in culture‐activated mouse HSCs treated with mAMSC‐122 supernatant. (**C**) Expression of mature Col1A1 decreased in mouse HSCs co‐cultured with mAMSC‐122. Blots against β‐actin served as loading controls. (**D** and **E**) MTT assay and PI staining were used to determine the cell proliferation and cell cycle of mAMSC‐122 supernatant‐treated mouse HSCs, respectively. Data are presented as means ± S.D. (**P < 0.01, *n* = 3).

We further analysed the effect of miR‐122‐modified mAMSCs on primary mouse HSCs. As shown in Figure 3A, miR‐122 was constitutively expressed in quiescent mouse HSCs. However, miR‐122 expression was significantly down‐regulated in mouse HSCs upon culture‐induced activation. Treatment of the culture‐activated mouse HSCs with mAMSC‐122 supernatant increased the miR‐122 level in the cells (Fig. 3B), also inducing significant cell growth inhibition (Fig. 3D) and G0/G1 arrest (Fig. 3E) in HSCs. Moreover, the protein level of Col1A1 significantly decreased in mAMSC‐122 co‐cultured HSCs similar to that in the hAMSC‐122 co‐cultured LX2 cells (Fig. 3C).

### Exosomes mediate miR‐122 communication between AMSCs and HSCs

**Figure 4:**
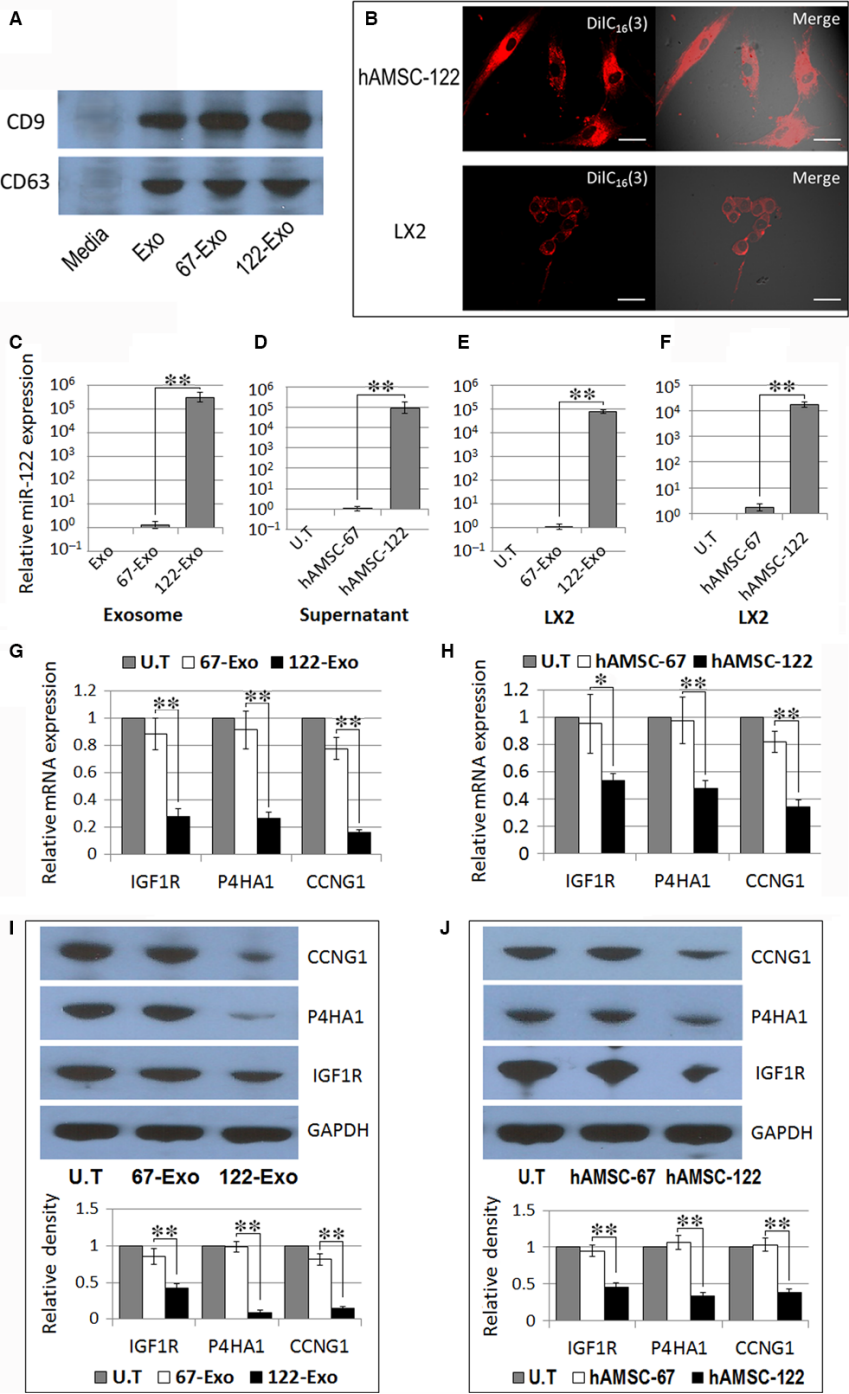
Exosome‐mediated miR‐122 communication between AMSCs and hepatic stellate cells (HSCs). (**A**) Western blot analysis of CD9 and CD63 expression levels in AMSC‐derived exosomes. (**B**) Confocal images of AMSC‐122 stained with DilC_16_(3), a phospholipid membrane dye. Transfer of fluorescent exosomes from AMSC‐122 was apparent in LX2 cell membranes and cytoplasma (×400). Scale bar = 30 μm. (C‒F) qPCR detection of miR‐122 expression in exosomes (**C**) or supernatant (**D**) from naïve hAMSCs (Exo), hAMSC‐122 (122‐Exo) or hAMSC‐67 (67‐Exo), and the exosome‐ (**E**) or hAMSC supernatant‐ (**F**) treated LX2 cells. (G‐J) qPCR and Western blot analysis of the expression levels of miR‐122‐targeted genes in LX2 cells treated with exosomes (**G**,** I**) or hAMSC supernatant (**H**,** J**). Blots against GAPDH served as loading controls. Data are presented as means ± S.D. (*P < 0.05, **P < 0.01, *n* = 3). U.T, untreated.

MiRNAs are abundant in extracellular exosomes and can be transferred from cell to cell *via* exosome release and uptake, resulting in cross‐cellular gene regulation 17. Increasing lines of evidence suggest that MSC‐derived exosomes play a role in the therapeutic effect of MSCs through paracrine mechanism. As shown in Figure 4A, MSC‐derived exosomes positively express of exosomal markers, such as CD9 and CD63. To assess the role of AMSC‐derived exosomes on miR‐122 communication, we labelled the miR‐122‐modified hAMSCs with lipophilic carbocyanine DilC_16_(3). After additional culture for 48 hrs, fluorescent exosomes were collected and added into the recipient LX2 cells. Confocal imaging revealed the delivery of labelled exosomes as indicated by the presence of fluorescent membrane dyes in the unlabelled recipient LX2 cells (Fig. 4B). Another proof is that the expression level of miR‐122 was 2.95 × 10^5^ and 7.24 × 10^4^‐fold higher in hAMSC‐122 exosomes (122‐Exo) and 122‐Exo‐treated LX2 cells than those in hAMSC‐67 exosomes (67‐Exo) and 67‐Exo‐treated LX2 cells, respectively (Fig. 4C and E). We also determined the miR‐122 levels in hAMSC supernatant and its supernatant‐treated LX2 cells; miR‐122 expression was significantly up‐regulated in both types of cells compared with that in hAMSC‐67 supernatant and its supernatant‐treated controls, respectively (Fig. 4D and F). These data indicated that AMSCs packaged miR‐122 into secreted exosomes, and these exosomes delivered miR‐122 into LX2 cells.

To further define the effects of exosome‐mediated miR‐122 communication on HSCs, we evaluated the expression levels of miR‐122 target genes, such as P4HA1, IGF1R and CCNG1 which are involved in the proliferation and collagen maturation of HSCs 11, 18, 19, in 122‐Exo‐treated LX2 cells. As shown in Figure 4G and I, treatment of LX2 cells with 122‐Exo significantly down‐regulated the mRNA and protein levels of these genes. We also investigated these genes in hAMSC supernatant‐treated LX2 cells, and the expression levels of these target genes were also significantly down‐regulated in hAMSC‐122 supernatant‐treated LX2 cells, compared with those in hAMSC‐67 supernatant‐treated controls (Fig. 4H and J). These data showed that miR‐122 modification improves the therapeutic potential of AMSCs against HSCs through exosome‐mediated miR‐122 communication.

### MiR‐122 modification improves the therapeutic potential of AMSCs against liver fibrosis

**Figure 5:**
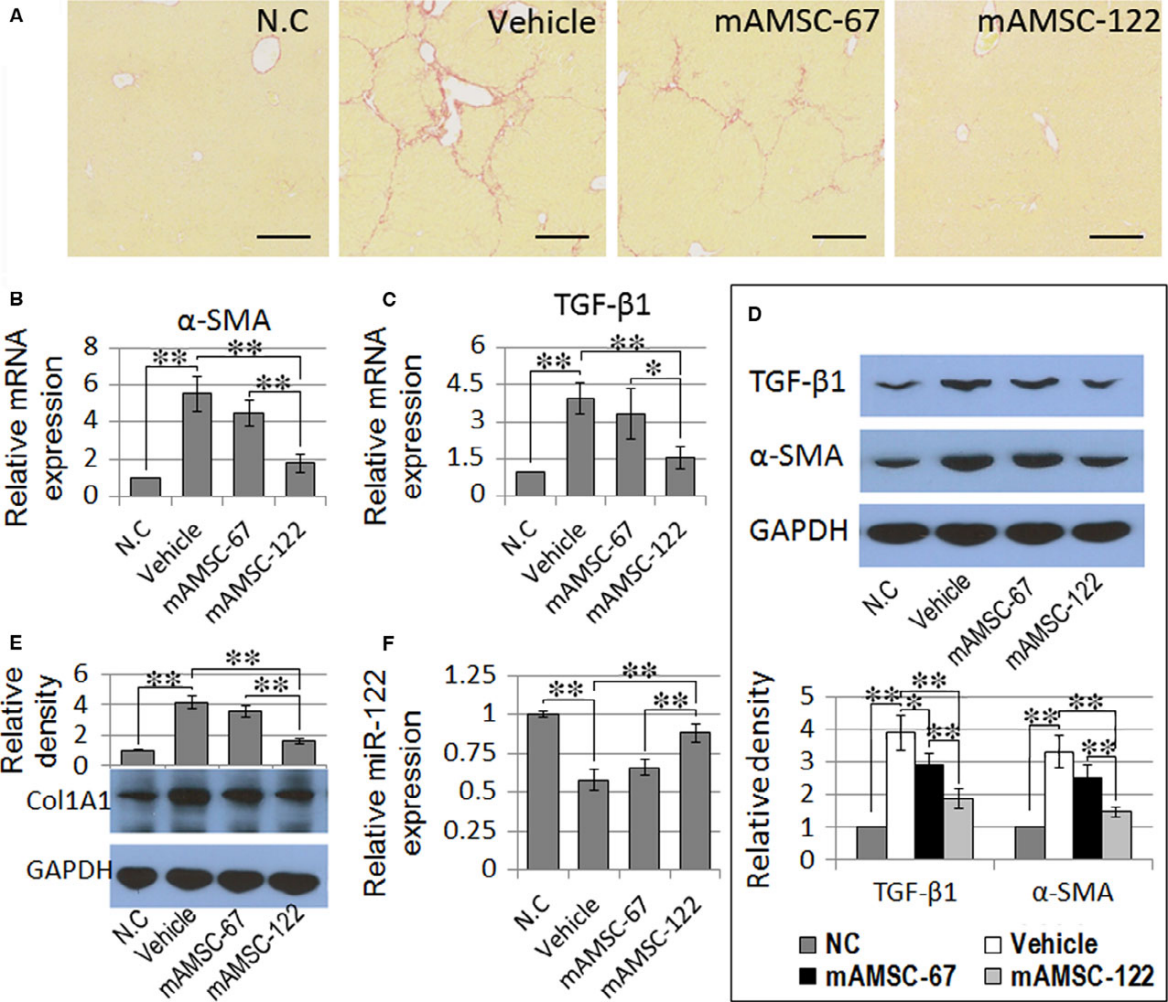
Effects of miR‐122‐modified AMSC transplantation on liver fibrosis. (**A**) Sirius red staining of the liver samples of vehicle, mAMSC‐67 and mAMSC‐122 administration groups at 6 weeks after CCl_4_ exposure (×200). Scale bar = 250 μm. (**B**–**D**) qPCR and Western blot analysis of α‐SMA and TGF‐β1 expression levels in liver samples. (**E**) Protein level of Col1A1 was examined through Western blot analysis. Blots against GAPDH served as loading controls. (**F**) qPCR detection of miR‐122 expression in liver samples. Data are presented as means ± S.D. (*P < 0.05, **P < 0.01, *n* = 5). N.C., normal control.

To further determine whether miR‐122‐modified AMSCs display better therapeutic abilities to reverse liver fibrosis, we administrated mAMSC‐122 or mAMSC‐67 into mice with chronic liver failure (2 weeks after CCl_4_ injection) *via* i.v. injection. Sirius red staining revealed that 6 weeks of exposure to CCl_4_ resulted in marked development of liver fibrosis. After administration of AMSCs for 4 weeks, the livers from the vehicle group showed formation of bridging fibrosis, whereas mAMSCs‐122 administration significantly suppressed fibrosis development. Moreover, the degree of liver fibrosis in mAMSC‐67 group slightly differed from that in the vehicle group (Fig. 5A).

The serum levels of HA, P‐III‐P, ALT, AST and liver hydroxyproline content were significantly suppressed in mAMSC‐122 administration group compared with those in the vehicle group (Table 1). The expression levels of TGF‐β1 and α‐SMA in the liver were also significantly down‐regulated in mAMSC‐122 group. However, these markers were only slightly suppressed in mAMSC‐67 group (Fig. 5B–D). Additionally, down‐regulation of miR‐122 in the mice liver as a result of repeated CCl_4_ treatment was ameliorated in mAMSC‐122 group compared with that in vehicle group. The level of mature Col1A1 protein was also significantly reduced in mAMSC‐122 group relative to that in the vehicle group. The levels of miR‐122 and mature Col1A1 proteins slightly differed between mAMSC‐67 group and vehicle group (Fig. 5E and F). These results indicated that miR‐122 modification enhanced the therapeutic efficacy of AMSCs against in liver fibrosis.

**Table 1:** MiR‐122‐modified mAMSC suppress serum fibrosis markers in CCl_4_‐induced liver fibrosis

	HA (ng/ml)	P‐III‐P (ng/ml)	ALT (U/ml)	AST (U/ml)	Hydroxyproline (μg/g)
N.C	26.2 ± 5.9† ^,^ ‡	8.2 ± 2.7† ^,^ ‡	32.4 ± 4.9† ^,^ ‡	81.8 ± 6.3† ^,^ ‡	205.7 ± 22.8† ^,^ ‡
Vehicle	196.6 ± 24.7¶	40.5 ± 4.1¶	189.8 ± 35.5¶	232.3 ± 34.7¶	567.3 ± 74.7¶
mAMSC‐67	141.8 ± 29.8¶	31.9 ± 5.3¶	131.5 ± 26.1¶	154.8 ± 22.9*^,^ ¶	460.4 ± 56.3¶
mAMSC‐122	41.2 ± 12.5† ^,^ ‡	16.3 ± 3.9† ^,^ ‡ ^,^ §	47.4 ± 12.2† ^,^ ‡	97.6 ± 21.5† ^,^ ‡	295.48 ± 48.5† ^,^ ‡ ^,^ §

Data are presented as the means ± S.D. HA, hyaluronic acid; P‐III‐P, procollagen III‐N‐peptide; ALT, alanine aminotransferase; AST, aspartate aminotransferase. *As compared with vehicle group, *P* < 0.05. ^†^as compared with vehicle group, *P* < 0.01; ^‡^as compared with mAMSC‐67 group, *P* < 0.01; ^§^as compared with N.C group, *P* < 0.05; ^¶^as compared with N.C group, *P* < 0.01.

## Discussion

Studies have shown that treatment of liver fibrosis with naïve MSC transplantation alone may be insufficient. Genetic manipulation, which aims to produce therapeutic genes, enhances the therapeutic abilities of MSCs 20, 21. MiR‐122 is down‐regulated in the livers of patients with primary biliary cirrhosis 22 and chronic HCV‐induced fibrosis 23, 24 and in the livers of mice treated with CCl_4_ 11, 12; moreover, miR‐122 plays a critical role in liver fibrosis by negatively regulating the proliferation and transactivation of HSCs 11. Thus, targeted delivery of miR‐122 into liver, particularly in HSCs, is a potential novel strategy for enhancing the therapeutic effect of MSCs on liver fibrosis. This study showed that miR‐122 modification in AMSCs improved the therapeutic potential of MSCs against liver fibrosis. In addition, AMSC‐derived exosomes may mediate the miR‐122 communication between AMSCs and HSCs, affecting the expression levels of miR‐122 target genes, which play a role in proliferation of and collagen maturation in HSCs.

Exosomes are small nanometer‐sized vesicles of endocytic origin; they essentially function as intercellular shuttle loaded with a cargo of protein and RNA by effector cells for off‐loading in target cells 17, 25, 26. Increasing studies indicate that MSCs demonstrate a scalable capacity to mass produce exosomes, which may play a role in therapeutic effect of MSCs through paracrine mechanism 27, 28, 29. Follow‐up studies have confirmed that MSC‐derived exosomes can be used as a vehicle for delivery of therapeutic miRNA 30, 31, 32. Our study revealed that miR‐122‐modified AMSC effectively packaged miR‐122 into secreted exosomes, which delivered their miR‐122 content into HSCs, resulting in altered miR‐122‐target gene expression in HSCs.

To understand the mechanism of miR‐122‐modified AMSCs in rescuing liver fibrosis, we examined the expression levels of miR‐122 target genes, which are involved in HSC proliferation and activation. Our results showed that IGF1R expression was significantly reduced by 122‐Exo treatment. Given that enhanced IGFR signalling is implicated in rapid proliferation of activated HSCs 33, 122‐Exo‐mediated down‐regulation of this gene may contribute to the inhibition of HSC proliferation by miR‐122‐modified AMSCs. A similar growth inhibitory effect of miR‐122 by regulation of several tumorigenesis‐related proteins, including ADAM10, TFDP2 and E2F1, was previously demonstrated in hepatoma cells 34, 35.

Cell cycle arrest contributes to growth inhibition. Reports have shown that MSCs exert a growth inhibitory effect on HSCs by inducing G0/G1 arrest *via* secretion of the soluble factors TGF‐beta3 and HGF, resulting in up‐regulation of p21 expression and down‐regulation of cyclin D1 36. Other studies have shown that ectopic expression of miR‐122 inhibits the growth of hepatoma cells. This finding is correlated with an increase in population of cells at the G1 phase and a decrease in that at the G2/M phase by modulating the expression of cyclin G1, which is involved in G2/M arrest in response to DNA damage 37. Our results showed that miR‐122‐modified AMSCs more effectively induced G0/G1 arrest of HSCs than scramble miRNA‐modified AMSCs. This finding was observed possibly because down‐regulation of 122‐Exo‐mediated CCNG1 reduced the cell population in G2/M phase, thereby enhancing the G0/G1 arrest of HSCs by miR‐122‐modified AMSCs.

In addition to its ability to inhibit proliferation, miR‐122 blocks collagen maturation in HSCs. P4HA1, which encodes a component of prolyl 4‐hydroxylase, a key enzyme in collagen post‐translational modification and maturation, is another target gene of miR‐122 11. We found that 122‐Exo treatment reduced P4HA1 expression level, and co‐culture with miR‐122‐modified AMSCs significantly inhibited the production of mature Col1A1 in LX2 cells. These data suggested that 122‐Exo‐mediated down‐regulation of P4HA1 contributed to the inhibitory effect of miR‐122‐modified AMSCs on maturation of collagen.

Systemically administered MSCs in animal models can engraft into the liver and ameliorate liver fibrosis caused by CCl_4_ 38, 39. However, some studies have shown that timing of MSC administration is an important determinant of the therapeutic effects of MSCs 4, 40. Enhanced liver function recovery was observed when MSCs were delivered at the onset of CCl_4_ injury and not under delayed administration. This phenomenon is possibly caused by the irreversible course of CCl_4_‐induced injury; wherein once injury occurred, it can no longer be rescued by MSC administration. Our study showed that administration of naïve AMSCs (data not shown) or scramble miRNA‐modified AMSCs 2 weeks after the first CCl_4_ injection did not effectively ameliorate liver fibrosis. While administration of miR‐122‐modified AMSCs successfully down‐regulated the serum fibrotic markers HA and P‐III‐P, reduced the serum ALT and AST levels and reduced collagen deposition in liver fibrosis.

To further investigate the underlying mechanism of miR‐122‐modified AMSCs in rescuing CCl_4_‐damaged liver, we examined the expression levels of TGF‐β1 and α‐SMA, which are key cytokines involved in the development of liver fibrosis and in HSC activation 41. We found that miR‐122‐modified AMSCs more effectively prevented the up‐regulation of TGF‐β1 and α‐SMA in fibrotic mouse livers than the scramble miRNA‐modified AMSCs. Moreover, the protein level of mature Col1A1 in AMSC‐122 administration group decreased, which is correlated with the increase in miR‐122 expression in the liver. These data suggest that AMSC‐122 treatment may ameliorate liver fibrosis by suppressing HSC activation. Studies have shown that miR‐122 expression decreases in advanced fibrosis and is negatively correlated with fibrosis stage and liver stiffness values in hepatic fibrosis with various aetiologies 12, 24, 42, 43; thus, the lower degree of reduction in miR‐122 level in the liver is possibly due to the amelioration of hepatic fibrosis by AMSC‐122 administration and not solely because of the delivery of miR‐122 into the liver. These results suggest that transplantation of miR‐122‐modified AMSCs presents several advantages over the transplantation of naïve AMSCs for treatment of liver fibrosis; these advantages include inhibition of HSC activation and blockade of collagen maturation. Thus, miR‐122 modification can broaden the time window for MSC transplantation.

In summary, miR‐122 modification can improve the therapeutic efficacy of AMSCs for the treatment of liver fibrosis. Administration of miR‐122‐modified AMSC not only repairs damaged liver; rather, AMSC‐derived exosomes mediate miR‐122 communication of donor cells to host HSCs and inhibit the proliferation and collagen maturation of HSCs, thereby reducing fibrotic changes in liver.

## Author's contributions

LG carried out the construction of miR‐122‐modified adipose tissue‐derived MSCs (AMSCs), performed the *in vivo* studies and drafted the manuscript. YY, LF, YB and CZ participated in the *in vitro* and *in vivo* experiments and performed the statistical analysis. ZM and LY conceived of the study and participated in its design and coordination. All authors read and approved the final manuscript.

## Conflict of interest

The authors confirm that there is no conflict of interests.
